# Synergistic Effect between Piperazine Pyrophosphate and Melamine Polyphosphate in Flame Retardant Coatings for Structural Steel

**DOI:** 10.3390/polym14183722

**Published:** 2022-09-06

**Authors:** Lianliang Li, Yating Huang, Wei Tang, Yi Zhang, Lijun Qian

**Affiliations:** 1 School of Artificial Intelligence, Beijing Technology and Business University, Fucheng Road No. 11, Haidian District, Beijing 100048, China; 2 China Light Industry Engineering Technology Research Center of Advanced Flame Retardants, Fucheng Road No. 11, Haidian District, Beijing 100048, China; 3 Petroleum and Chemical Industry Engineering Laboratory of Nonhalogen Flame Retardants for Polymers, Fucheng Road No. 11, Haidian District, Beijing 100048, China; 4 College of Chemistry and Materials Engineering, Beijing Technology and Business University, Fucheng Road No. 11, Haidian District, Beijing 100048, China

**Keywords:** intumescent flame retardant, synergistic effect, intumescence coating (IC), structural steel

## Abstract

Piperazine pyrophosphate (PAPP) combined with melamine polyphosphate (MPP) was adopted to prepare a waterborne fire retardant intumescent coating (IC) for structural steel. Silicone acrylic emulsion was used as binder. In the 2-h torch test, PAPP/MPP-IC coating presented excellent fire resistance performance. The equilibrium temperature at the backside of the steel board decreased to 170 °C with protection of MPP/PAPP-IC, compared with 326 °C of APP/PER/MEL-IC. After 72-h water immersion, MPP/PAPP-IC could still provide sufficient thermal isolation, but APP/PER/MEL-IC failed the test. The water absorption of the MPP/PAPP coating was also reduced. The thermogravimetric analysis measured that the PAPP/MPP-IC had unique initial decomposition temperature of 296 °C and higher residue of 33.8 wt%, which demonstrated better thermal stability and fire retardancy in condensed phase. In addition, Scanning Electron Microscope (SEM) images illustrated that the structure of the carbon layer formed by MPP/PAPP-IC was dense, complete and consistent, indicating the improvement of mechanical strength and thermal isolation of the char. The synergistic effect between piperazine and phosphoric acid groups in MPP/PAPP contributed to the superior flame retardancy. Consequently, MPP/PAPP-IC was much more efficient than the traditional APP/PER/MEL-IC. This work provides a novel way for designing flame retardant coatings for structural steel with excellent comprehensive performance.

## 1. Introduction

Structural steel is one of the most widely used materials for building construction. It is a non-combustible material that shows good ductility. However, it loses almost 40–45% of its strength once the temperature reaches above 500 °C [1,2]. Hence, it is crucial to protect structural steel from collapsing in fire, thus guarantee the safety of people.

Intumescence coating (ICs) have been gaining much attention because they are applied on structural steel as fire protection [1]. Intumescent flame retardant (IFR) is an important part of IC. The IFR system consists of three components: acid source (dehydrating agent, such as ammonium polyphosphate (APP)), carbon source (char forming agent, such as pentaerythritol (PER)), and blowing agent (such as melamine (MEL)). A swelling char was generated at high temperature to inhibit the heat transfer to the structural steel. As a traditional IFR system, the composition of APP/PER/MEL was commonly used in ICs. However, due to the high polarity of the components, APP/PER/MEL system is humidity sensitive [3]. In practical application, the coatings often suffer from the erosion of water and moisture [4]. The hydrophilic APP is easy to migrate after water immersion, leading to the failure of fire protection [5,6]. Thus, it is necessary to investigate water resistant ICs.

Considerable efforts have been made to overcome the disadvantages of traditional IFRs and improve water resistance of ICs. The surface grafting kinetics and experimental results indicated that surface modification of APP with MEL can be helpful [7]. Some of the ammonium cations in APP was replaced by melamine, which will increase its water resistance. On the other hand, the microencapsulation of flame retardants has satisfactory results. Sun et al. found that co-microencapsulating APP and PER greatly improved the fire resistance and thermal stability of epoxy coatings [8]. Moreover, novel macromolecular flame retardants with acid source, carbon source and blowing agent have been synthesized, such as melamine polyphosphate (MPP) [9], piperazine pyrophosphate (PAPP) [10], charring foaming agents (CFA) [11], caged bicyclic phosphate (Trimer) [12] et al. They suppress the deterioration of hygroscopicity, decrease FR exudation and improve system compatibility.

PAPP is prepared by copolymerization of piperazine and phosphoric acid [13], which has been successfully applied in polypropylene [10,14], thermoplastic elastomer [15] and polyamide 66 [16]. PAPP, containing the three indispensable components of IFR in the molecular structure simultaneously, owns high initial decomposition temperature and superior charring ability. In addition, PAPP shows excellent water resistance due to its macromolecular structure. MPP can act as an acid source and a blowing agent. It can not only catalyze the formation of the protective char layer, but also release non-flammable gases like NH_3_ [16]. Liang et al. [17] used MPP as intumescent flame retardant in acrylic resin to remarkably enhance its flame retardancy and heat resistance. The limiting oxygen index (LOI) value raised to 30% and the heat resistance index (THRI) was 189.1 °C. The interaction between PAPP and MPP accelerates the formation of the high-quality char layer. The composites containing PAPP and MPP increased the LOI to 39.9% and 37.8% for glass fiber polypropylene [10] and thermoplastic elastomer [15], respectively. To data, synergistic effect between MPP and PAPP in ICs for structural steel has been seldom reported.

Silicone acrylic emulsions (SAE) are widely used as binders in coatings because of its excellent weatherability, aging resistance and hydrophobicity [18]. Titanium dioxide (TiO_2_) is commonly used as white pigment in coating industry. It has been reported that at high temperature TiO_2_ reacted with APP or its degradation products, such as polyphosphoric acids, and/or phosphorus pentoxide pyrophosphate to form titanium pyrophosphate (TiP_2_O_7_), which was thermally stable and decreased the radiative heat transfer from the surface thereby improving the thermal insulation properties [19]. Chen et al. [15] illustrated that the interaction between PAPP/MPP/TiO_2_ and TPE during decomposition process induced the formation of crosslinking residue, and its char formation rate reached 23.5 wt% and exhibited good thermal stability.

(1) $$2{\mathrm{TiO}}_{2}+{\left({\mathrm{NH}}_{4}\right)}_{4}P_{4}O_{12}\to 2{\mathrm{TiP}}_{2}O_{7}+4{\mathrm{NH}}_{3}↑+2H_{2}O↑$$

This paper evaluated the synergistic effect and mechanism between MPP and PAPP in SAE intumescence coatings for structural steel. TiO_2_ was adopted as fillers. APP/PER/MEL-IC was the control group. Fire protection performance, water resistance properties and thermal stability of the coatings were evaluated. This study will deepen the understanding of the fire resistance and thermal insulation of protective coatings with different flame retardant systems, and provide experimental evidence for the development of water resistance fireproof coatings.

## 2. Methods

### 2.1. Materials

Silicone acrylic emulsion (SAE, Hehe Chemical Technology Co., Ltd., Shanghai, China) with a solid content of 50 ± 3% was used as the binder. The content was 65 wt% for both formulas. Titanium dioxide (TiO_2_, rutile, Macklin Biochemical CO., Ltd., Shanghai, China) was added as the filler. The content was 5 wt%.

The formulas were listed in Table 1. APP (polymerization degree *n* > 1000) were purchased from JLS Flame Retardants Chemical Co., Ltd., Hangzhou, China. PER was supplied by Sino pharm Chemical Reagent CO., Ltd., Beijing, China. MEL was obtained from Macklin Biochemical CO., Ltd., Shanghai, China. MPP (NP-200) was provided by Shouguang Weidong Chemical Co., Shouguang, China. PAPP was purchased from Kejufu New Material Co. Ltd., Chongqing, China.

### 2.2. Sample Preparation

Q235 steel (10 cm × 10 cm × 1 cm) and stainless steel (10 cm × 10 cm × 0.2 cm) were used as substrates, and all the steel plates was polished, cleaned and dried for use. The stainless steel was used for water immersion test.

**Figure 1:**
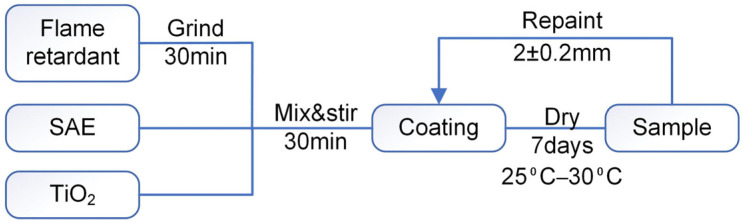
Flow chart of coating preparation.

The coating preparation process was shown in Figure 1. The IFR mixture was ground for 15 min. Then, the flame retardant (30 wt%) and the titanium dioxide (5 wt%) were added to the silicone acrylic emulsion (SAE, 65 wt%) and mixed by a high speed blender at 350 rpm for 15 min at 25 °C. The prepared coating was painted on steel plates and dried in the oven at 30 °C. This process was repeated 3–5 times. The coating samples were cured in a dry and well ventilated environment for 7 days. Finally, the film thickness reached 2 ± 0.2 mm.

### 2.3. Fire Performance

**Figure 2:**
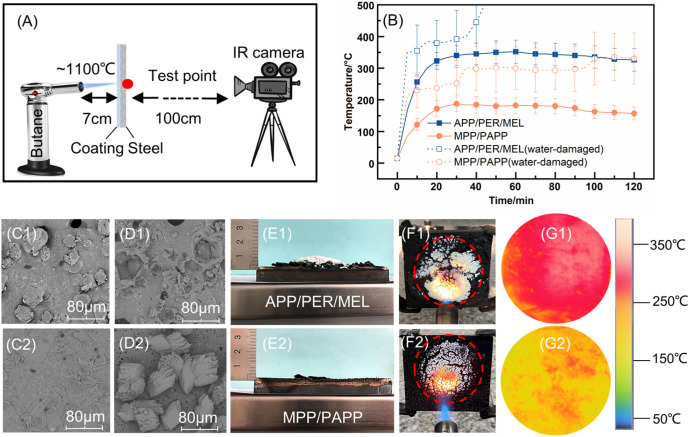
(**A**) Schematic representation of the fire performance test. (**B**) The backside temperature curves of steel boards with coating samples in the torch test. (**C**) SEM images of the coating surface. (**D**) Surface morphology of coatings after 72-h water immersion. (**E**) Height of swelling chars after the torch test. (**F**) Digital pictures of char layer at 120 min in the torch test. (**G**) Thermal maps on the backside of the steel boards at 120 min in the torch test. **C1**–**G1** were for APP/PER/MEL-IC, **C2**–**G2** were for MPP/PAPP-IC.

Fire endurance of the coating was evaluated using a homemade fire testing method based on the torch test (Figure 2A). According to the Big panel method (GB/T12441–2005 in China), the coating was exposed to an open flame (temperature of the flame around 1100 °C). The gas consumption of the butane cylinder was 160 g/h. The distance between the center and the sample was 7 cm, and the thermal maps at the backside of the steel plates were captured as a function of time using a thermal imaging camera (FLIR T660, FLIR Systems, Nashua, NH, USA). The temperature was measured in the circular area with the diameter of 10 cm. The value was recorded every 5 min thereafter until the highest temperature reached 400 °C [20], or the duration time reached 120 min. This test allowed the evaluation of the fire protection performance of an intumescent coating in a convective heating scenario

In addition, the intumescent factor is calculated as follows:

(2) $$\mathrm{IF}=\frac{d_{2}}{d_{1}}$$

where d_1_ denoted the thickness of the unexpanded coating, d_2_ denoted the swelling char thickness. The original coating thickness was 2 mm.

### 2.4. Water Resistance

The static immersion test was carried out to assess the water-resistance of the coating. The stainless steel plates covered with coatings were immersed in distilled water for 72 h at 25 °C and then dried with a piece of paper towel to remove excess water. The weight of the coating samples was measured at different immersion time. The weight change of the samples was calculated using the following method [21]:

(3) $$\Delta w=\frac{w_{e}-w_{0}}{w_{0}}\times 100\%$$

where Δw is the weight change ratio of the coating; $w_{e}$ is the weight of the coating after water immersion; and $w_{0}$ is the weight of the coating before water immersion.

### 2.5. Thermal Stability

Thermal stability was analyzed by the simultaneous thermal analyzer (STA 8000 PerkinElmer, Waltham, MA, USA). The coating sample of 2~4 mg was placed in an alumina crucible and heated from 50 °C to 800 °C at the rate of 20 °C/min in N_2_ atmosphere.

### 2.6. Contact Angle Analysis

The water contact angle (CA) was measured by a CA goniometer (OCA35, Data physics Company, Filderstadt, Germany). The powder samples (30 g) were pressed to tablets with diameters of 50 mm and height of 3 mm. The droplet was 2 μL. Five different points were taken for each sample.

### 2.7. Infrared Spectrum Analysis

The molecular structure during carbon formation was characterized by an FTIR spectrometer (Nicolet in 10MX, Thermo Scientific, Madison, WI, USA). The potassium bromide (KBr) disk (containing 0.5 mg testing samples and 50 mg KBr) was used for detection. The resolution is 128 scans per centimeter (128 cm^−1^).

### 2.8. Morphology Characterization

The expansion of the char layer was captured by a digital camera and the height was measured in millimeters. Scanning electron microscopy (SEM, FP 2032/14 Quanta 250FEG, Phenom World, Eindhoven, The Netherlands) was used to observe the micrometer structure of the coating surface and the char on the micron scale. The acceleration voltage was 20 kV. Gold spray was required for the insulating char layer.

## 3. Results and Discussion

### 3.1. Fire Protection of the Coatings

MPP/PAPP-IC showed perfect flame retardancy, which were competitive in comparison with existing fire-retardant coatings for steel in literatures using APP/PER/MEL or APP/EG/MEL systems, as shown in Table 2. The duration time was the longest of 120 min. The load of flame retardant was as usual. The equilibrium temperature was much lower than that in most of the reports.

The time-temperature curves on the backside of steel plates coated with different IC formulations were plotted in Figure 2B. The solid lines stood for the coatings before the water immersion test. The equilibrium temperature of undamaged MPP/PAPP-IC (orange color) was only 170 °C during the 2-h test. This was remarkably lower than the equilibrium temperature of 326 °C for APP/PER/MEL-IC. The surface temperature was also more uniform for MPP/PAPP-IC, based on the error bars and the thermal map in Figure 2G.

Furthermore, the water-damaged MPP/PAPP-IC (dash line in orange in Figure 2B) can pass the fire performance test, although the equilibrium temperature raised to 334 °C. On the contrary, APP/PER/MEL-IC failed the test. The backside temperature exceeded 400 °C in 40 min.

The profiles of time-temperature curves of two coatings displayed similar shapes, but the equilibrium temperature of undamaged MPP/PAPP-IC was much lower. During the first 20 min, the thermal conductivity of the steel is high and the coating was thin. The steel backside temperature increased rapidly to about 200 °C. The chemical reactions started, allowing the formation of the swelling char layer. The thermal conductivity decreased because of the porous structure and the increased thickness of the char. Thereby, the temperature rising begun to slow down.

Intumescent char layer after the torch test were presented in Figure 2E,F, with maximum thickness and IF of the swelling char in Table 3. The MPP/PAPP-IC formed a denser and stronger carbon layer as shown in Figure 2F2, which inhibited heat transfer from fire to the steel substrate, although its intumescent factor was lower than that of APP/PER/MEL-IC (Table 3) [1]. In addition, the distribution of the white mineral material was more uniform for MPP/PAPP-IC, which should be a mixture of TiP_2_O_7_ and TiO_2_ [32], as shown in Figure 2F. This white shield would contribute to the drop down of the equilibrium temperature of MPP/PAPP-IC. As a result, MPP and PAPP in SAE coating presented good synergistic effect.

### 3.2. Water Resistance of the Coatings

After the water immersion test, the fire retardancy of coatings decreased as shown in Figure 2B. The APP/PER/MEL-IC broken down at 40 min and the steel back side temperature went up to 400 °C shortly. Although MPP/PAPP-IC was also affected by water, it still passed the torch test. The highest temperature was kept lower than 400 °C. The weight change rate of the coatings in water immersion test, contact angles of ICs and the flame retardants and surface topology of the coatings were examined to understand the reason why MPP/PAPP-IC had better water resistance.

**Figure 3:**
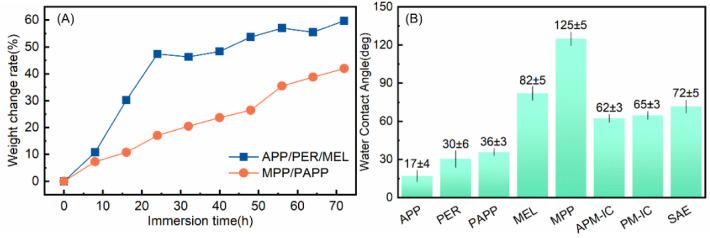
(**A**) The weight change of the coatings immersed in distilled water. (**B**) Water contact angle of IFR and ICs.

To evaluate the water adsorption of the coating, the weight change of the coatings immersed in water for different time was compared. Results were plotted in Figure 3A. It showed that the weight of both coatings increased gradually with longer immersion time, as the permeation process of water into the coatings exceeded the migration process of fire retardant ingredients [33]. MPP/PAPP-IC displayed lower weight change which suggested that it had better water resistance than APP/PER/MEL-IC.

The absorbing water would destroy the crosslinking structure of the coating and might cause the coating to flake off [33]. SEM images of the coating surface showed the difference before and after water immersion (Figure 2C,D). The prepared coatings without water immersion were illustrated in Figure 2C. The surface of MPP/PAPP-IC was smoother with less particle defects, which demonstrated that the MPP/PAPP system had better compatibility in SAE binder, leading to the reduction of water seepage. Furthermore, on the surface of water-damaged APP/PER/MEL coating, it can be observed clearly that there were cavities left by the dissolved APP, which was the key active ingredient. While the flame-retardant crystal was left on the surface of the coating, although it migrated.

Hydrophobic surface will prevent the water adsorption. APP/PER/MEL-IC had poor water resistance owing to the super hydrophilic APP and PER. In contrast, MPP/PAPP-IC consisted of more hydrophobic components. The contact angles were compared in the bar chart in Figure 3B. The contact angle of MPP was 125°, which was the largest. The contact angle of PAPP was 36°, which was also larger than the contact angle of APP (only 17°). The contact angles of the coatings were 62° and 65° for the APP/PER/MEL-IC and MPP/PAPP-IC, respectively. The narrowing down of the hydrophobicity difference may be related to the effect of SAE binder (contact angle of SAE was 72°) and the different surface topology.

Accordingly, the fire resistance of the APP/PER/MEL-IC was seriously reduced after water immersion. MPP/PAPP-IC was better due to the lower water adsorption and lower flame retardant loss.

### 3.3. Thermal Analysis of the Coatings

**Figure 4:**
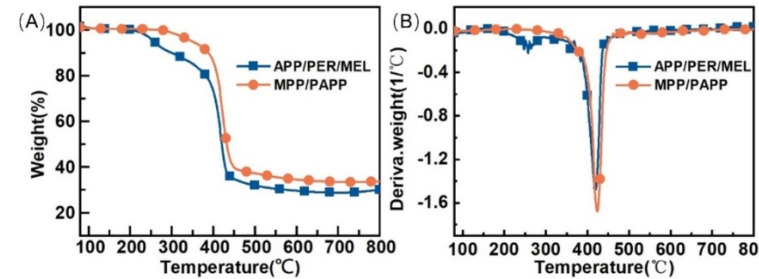
(**A**) TGA curves of coating samples. (**B**) DTG curves of coating samples.

As a fire-retardant coating, it is vital to assess its thermal stability. TGA results displayed that the coating with MPP/PAPP exhibited higher thermal stability and char yields than APP/PER/MEL-IC (Figure 4; Table 4) in N_2_ conditions. MPP/PAPP-IC started to decompose at 296 °C. This initial degradation temperature (T_d,1%_) was higher than APP/PER/MEL system, whose T_d,1%_ was 222 °C.

MPP/PAPP-IC presented a one-step decomposition behavior. There was a huge weight loss temperature from 380 °C to 450 °C. These results suggested that the IFR system of MPP, PAPP and TiO_2_ reacted simultaneously. PAPP, as a mono-component IFR, will decompose into piperazine [15], (PON)m, CO_2_, NH_3_, etc. MPP will release (HPO_3_)_n_ and incombustible gases, such as NH_3_ and H_2_O [16]. (HPO_3_)_n_ acted as the acid source and piperazine acted as the carbon source. Meanwhile, the esterification reaction between acid source and carbon source lead to the dehydration of carbon source to form a molten carbon layer [34]. The earlier blowing of gases would help to generate the porous char layer at lower temperature.

In addition, the reaction between TiO_2_ and degradation products of PAPP would generate TiP_2_O_7_, thereby building a hard and porous shield which effectively protected the char residue. This could increase the residue in the condensed phase. The residual carbon rate of MPP/PAPP-IC reached up to 33.8%, which was one of the reasons for the excellent fire resistance performance.

APP/PER/MEL-IC exhibited two-step decomposition behavior. The first decomposition stage occurred at about 230–350 °C, APP and PER decomposed [35]. The second stage of thermal decomposition occurred at about 380–450 °C, which was ascribed to the further pyrolysis of MEL. Unlike MPP/PAPP, the three components in APP/PER/MEL mixture did not decompose in the same temperature region. Although the delayed degration of the blowing agent was beneficial to the formation of a higher expanded carbon layer (as the results shown in Table 3), the thermal decomposition of other materials would significantly reduce the mass of the residue. The residual carbon rate of APP/PER/MEL-IC was only 29.9%, thus the flame retardant performance of the coating was affected [3]. Besides, the pressure caused by the gas inside the char would broke its structure when the molten carbon solidified. Therefore, the synergistic effect between MPP/PAPP was better.

### 3.4. Microstructure of the Intumescent Char Layers

**Figure 5:**
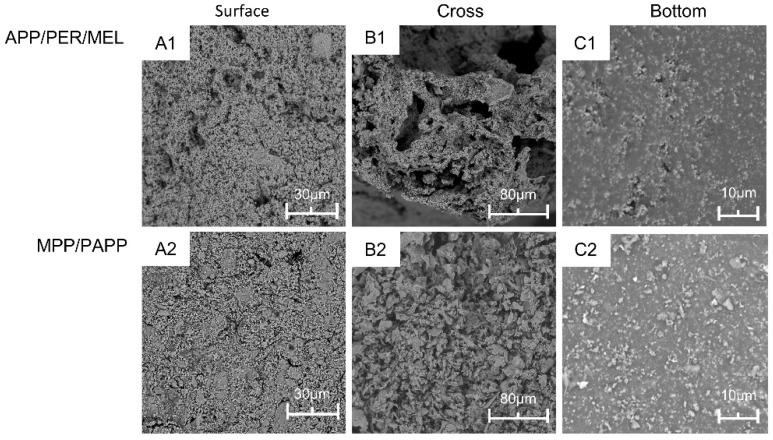
SEM images of the surface (**A1**,**A2**) layer, the cross-view (**B1**,**B2**), and the bottom (**C1**,**C2**) layer of the chars.

Generally, the structure and morphology of the swelling char layer have significant impact on the flame retardancy of the material [36]. SEM images of the swelling char after the torch test were shown in Figure 5. The pictures were taken from the top view of the surface, the cross section of the char and the bottom layer of the char adhering on the steel substrate.

The top surface of the char formed by MPP/PAPP-IC was denser with less defects than that formed by APP/PER/MEL-IC. There were cavities and cracks in Figure 5A1. These cracks would propagate with the gas released by the flame retardants inside and the impact of the flame outside, providing passage for heat and mass transfer. On the contrast, the surface of the MPP/PAPP-IC was more consistent and uniform. The combination of MPP/PAPP and TiO_2_ could form a stronger protective shield to block the flame and heat out of the char layer [15].

The inner structure of char layers could be observed from the cross section in Figure 5B. It was obviously that the char of MPP/PAPP-IC was denser and more uniform. The cross-linked carbon skeleton was strongly built and strengthened by adhesion TiO_2_ particles. Pores were finely and evenly distributed. Instead, the char layer of APP/PER/MEL-IC was made of layered framework with large cavities. The internal stress was much higher for such uneven structure, resulting in the collapse of the char (Figure 2F). Moreover, heat from the flame could pass through these cavities, resulting in an increase of the thermal conductivity.

The char layers at the bottom were continuous with embedded particles as shown in Figure 5C. The crosslinking structure of the bottom layer would facilitate the adhesion of the char layer. The bottom layer of MPP/PAPP-IC was more uniform with better dispersed fine particles. With the protection of the upper expansion layer, the temperature at the bottom layer should be much lower. The combination of carbon lava and unreacted coating material might be helpful to build such reliable adhesive layer. The backside temperature of the steel protected by MPP/PAPP-IC was lower, which may have contributed to the saving of coating material such as SAE, leading to the formation of the crosslinking structure.

As a result, MPP/PAPP-IC formed a hard, consistent and dense carbon layer during the fire. This was another reason for the high fire protection of MPP/PAPP-IC.

### 3.5. Analysis of the Flame Retardant Mechanism

To understand the decomposition process of the flame retardant coating, and further explore the synergistic effect between MPP and PAPP, the residues of the MPP/PAPP-IC at different temperatures were characterized by FTIR. Based on the decomposition behavior of MPP/PAPP-IC in Figure 4A, the selected temperature included room temperature (RT, 25 °C), 400 °C, and 500 °C.

**Figure 6:**
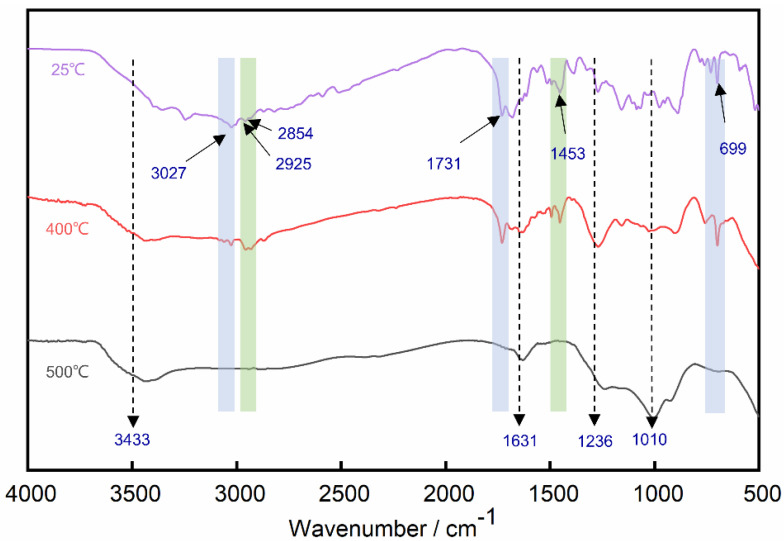
FTIR spectra of MPP/PAPP-IC at different temperatures.

The corresponding spectra were presented in Figure 6. The peak positions with the vibration modes were listed in Table 5.

The absorption peaks caused by typical structures in SAE matrix and PPAP/MPP system can be observed in room temperature. The peaks at 3027 cm^−1^ and 699 cm^−1^ were created by unsaturated =C-H structure in SAE molecule. The absorption peak of unsaturated C=C structure can be also found at 1631 cm^−1^. The absorption peaks at 2854 cm^−1^, 2925 cm^−1^ and 1453 cm^−1^ were brought in by saturated C-H groups in SAE matrix. The peak at 1731 cm^−1^ was contributed by the C=O group of acrylic acid skeleton in SAE.

The curve at 25 °C exhibited obvious absorption area in the range from 3000 cm^−1^ to 3700 cm^−1^, which were mainly caused by the N-H in piperazine group and NH_2_ in melamine component. The information of P=O and abundant PO_3_ structures in MPP and PAPP can be observed at 1236 cm^−1^ and 1010 cm^−1^, respectively. With the temperature increasing from 25 °C to 400 °C, it can be easily found that the absorption peaks of SAE matrix were significantly weakened, whereas the absorption peaks of phosphorus-containing structure became stronger. The results indicated that MPP/PAPP exerted flame retardancy in condensed phase, to form a porous and solid char layer with good thermal insulation in fire. The -NHx groups were more inclined to release incombustible gases to exert the dilution effect in the gas phase. Polyphosphate was generated and remained in the condense phase, which can be proved by the absorption peak at 3433 cm^−1^.

With the temperature rising to 500 °C, most of the absorption peaks disappeared comparing with the room temperature. The absorption peaks at 1631 cm^−1^ of C=C bonds indicated the formation of graphitization char. The peaks at 1010 cm^−1^ proved that the phosphoric acid and piperazine groups in the MPP/PAPP mixture reacted to generate melamine polyphosphates with branched or cross-linked structures. Besides, PO_3_ can be saved in the condensed phase as the TiP_2_O_7_ in the white shield (Figure 2F), by the reaction between and TiO_2_ and (HPO_3_)_n_. According to the above analysis, it can be concluded that the char layer of MPP/PAPP consisted of phosphorus-containing structure and the graphitized carbon [7]. Therefore, the synergistic effect between MPP and PPAP enhanced the charring capability of the IC, leading to better thermal insulation of the protection layer.

Consequently, the synergistic flame retardant effect between MPP and PPAP was reflected in the condensed phase and the gas phase. In the condensed phase, the chemical reaction between PAPP and MPP could accelerate the formation of dense and thermally stable char layer. The combination of flame retardant with TiO_2_ and SAE further enhanced the strength of the surface layer of the char. It was built as a barrier which played a key role in enforcing the fire resistance and thermal stability of ICs. In the gas phase, the released non-flammable gases such as NH_3_ and H_2_O could act as flame arrestors to terminate the combustion process and swell the char layer to enhance the thermal insulation of ICs for structural steel.

## 4. Conclusions

In this research, new waterborne intumescent paint for structural steel with MPP/PAPP as flame retardancy was developed. Fire protection performance, water resistance properties and thermal stability of the coatings were evaluated. The thermal insulation and flame retardant mechanism were analyzed via the characterization of the residual char. Findings were concluded as follows:

1. MPP/PAPP-IC displayed perfect fire resistance and thermal properties. The equilibrium temperature in torch test was only 170 °C in 2 h, which was remarkably lower than APP/PER/MEL-IC and literature reports. The residue reached up to 33.8 wt% in TGA and the initial decomposition temperature was higher, indicating better thermal stability.
2. The water-damaged MPP/PAPP-IC could still pass the fire resistance test. The ingredients of MPP/PAPP had better compatibility in SAE binders, and MPP and PAPP were more hydrophobic than APP, leading to the reduction of water absorption. Therefore, the water resistance of the coating was improved.
3. MPP/PAPP-IC exhibited great charring capability through the reaction between phosphoric acid and piperazine groups. Reaction between (HPO_3_)_n_ and TiO_2_ further enhanced the strength of the surface layer of the char. More uniform and denser char structure was generated, which inhibited the heat transmission, thus improved fire proof properties.

The findings provided evidence for the synergistic flame retardant effect between phosphoric acid and piperazine groups in the MPP/PAPP mixture, which contributed to the formation of thermal insulation char layer for structural steel. It gave implications for the development of ICs in the future. Further research should be undertaken to continually improve the water resistance of the coating.

## Figures and Tables

**Table 1:** Formulations of IFR.

Sample	IFR (wt%)
APP/PER/MEL	15/6/9
MPP/PAPP	10/20

**Table 2:** Comparison of fire performances of as-designed fire retardant ICs for structural steel with previous counterparts.

Fire Retardant	Binder	Filler	Time/Min	Equil. Temp./°C	Ref
MPP/PAPP (30 wt%)	SAE	TiO_2_ (5 wt%)	120	170	This work
MF-APP/PER/MEL ^#^ (54.2 wt%)	Acrylic resin	MF-BZ ^#^ (6 wt%)	100	212	Huo [4]
APP/PER/MEL (37 wt%)	VAC *	CaCO_3_ (10 wt%)	100	264	Md Nasir [22]
APP/PER/MEL (40 wt%)	VAC *	TiO_2_/BioAsh (10 wt%)	60	113	Beh [23]
APP/PER/MEL (40 wt%)	Acrylic resin	TiO_2_ (2.4 wt%)	60	171	Wang [24]
MPP/DPER/MEL (25 wt%)	Epoxy	CNP@Mo ^‡^ (2 wt%)	60	180	Xiao [9]
APP/PER/MEL (37 wt%)	Acrylic resin	TiO_2_/Mg(OH)_2_ (7.4 wt%)	60	188	Yew [25]
APP/PER/MEL (45 wt%)	VAE *	Na-REC ^‡^/TiO_2_ (15 wt%)	60	202	Xie [26]
APP/EG/MEL ^#^ (23 wt%)	Epoxy	Boric Acid/Kaolin clay (16.5 wt%)	60	257	Ullah [27]
APP/PER/MEL (50 wt%)	Acrylic resin	Nano-TiO_2_ (20 wt%)	60	289	Beheshti [28]
APP/EG/MEL ^#^ (23 wt%)	Epoxy	Boric acid (11 wt%)	60	337	Ullah [29]
APP/PER/EG ^#^ (27 wt%)	SAE	Al (OH)_3_ (3 wt%)	60	350	Zhou [30]
APP/PER/MEL (46 wt%)	Epoxy	TiO_2_ (10 wt%)	46	417	Tang [31]

* VAC: vinyl acetate copolymer; VAE: vinyl acetate-ethylene. ^#^ MF: melamine formaldehyde; EG: expandable graphite. ^‡^ CNP: carbon nitride polydopamine; BioAsh: from rubberwood; REC: rectorite.

**Table 3:** Information on swelling char.

	APP/PER/MEL	MPP/PAPP
Char thickness	14 mm	6 mm
Intumescent factor	7	3

**Table 4:** Thermogravimetric data of the flame retardant coatings in N_2_.

Coating	T_d,1%_ (°C)	R_800°C_ (wt%)
APP/PER/MEL	222	29.9
MPP/PAPP	296	33.8
MPP	379	28.9
PAPP	313	22.9

**Table 5:** The assignments of the characteristic vibration peaks of MPP/PAPP-IC [19,32].

	Band Position (cm^−1^)		
Assignment	25 °C	400 °C	500 °C
N-H stretching mode	-	3433	3433
C-H stretching mode	3027, 699	3027, 699	-
C=H stretching mode	2854, 2925, 1453	2854, 2925, 1453	-
C=O stretching mode	1731	1731	-
C=C stretching mode	-	1631	1631
PO_2_/PO_3_ stretching mode	-	1010	1010

## Data Availability

Data available on request from the authors.

## References

[1] Yasir M., Ahmad F., Yusoff P.S.M.M., Ullah S., Jimenez M. (2019). Latest trends for structural steel protection by using intumescent fire protective coatings: A review. Surf. Eng..

[2] Lazar S.T., Kolibaba T.J., Grunlan J.C. (2020). Flame-retardant surface treatments. Nat. Rev. Mater..

[3] Anees S.M., Dasari A. (2017). A review on the environmental durability of intumescent coatings for steels. J. Mater. Sci..

[4] Huo S., Wang C., Hu Q., Liu S., Zhang Q., Liu Z. (2020). A facile strategy to fabricate an intumescent fire-retardant coating with improved fire resistance and water tolerance for steel structure. J. Coat. Technol. Res..

[5] Jimenez M., Bellayer S., Revel B., Duquesne S., Bourbigot S. (2013). Comprehensive Study of the Influence of Different Aging Scenarios on the Fire Protective Behavior of an Epoxy Based Intumescent Coating. Ind. Eng. Chem. Res..

[6] Zhong S., Li J., Cai Y., Yi L. (2018). Novel surfactant-free waterborne acrylic-silicone modified alkyd hybrid resin coatings containing nano-silica for the corrosion protection of carbon steel. Polym. Technol. Mater..

[7] Cao K., Wu S.-L., Wang K.-L., Yao Z. (2011). Kinetic Study on Surface Modification of Ammonium Polyphosphate with Melamine. Ind. Eng. Chem. Res..

[8] Sun L., Qu Y., Li S. (2012). Co-microencapsulate of ammonium polyphosphate and pentaerythritol in intumescent flame-retardant coatings. J. Therm. Anal..

[9] Xiao G., Yang Z., Chen C., Chen C., Zhong F., Wang M., Zou R. (2021). Novel carbon nitride@polydopamine/molybdenum disulfide nanoflame retardant improves fire performance of composite coatings. Colloids Surf. A Physicochem. Eng. Asp..

[10] Yuan Z., Wen H., Liu Y., Wang Q. (2020). Synergistic effect between piperazine pyrophosphate and melamine polyphosphate in flame retarded glass fiber reinforced polypropylene. Polym. Degrad. Stab..

[11] Yang R., Ma B., Zhao H., Li J. (2016). Preparation, Thermal Degradation, and Fire Behaviors of Intumescent Flame Retardant Polypropylene with a Charring Agent Containing Pentaerythritol and Triazine. Ind. Eng. Chem. Res..

[12] Shi Y., Wang G. (2016). The novel silicon-containing epoxy/PEPA phosphate flame retardant for transparent intumescent fire resistant coating. Appl. Surf. Sci..

[13] Xu M.-J., Xia S.-Y., Liu C., Li B. (2018). Preparation of Poly(phosphoric acid piperazine) and Its Application as an Effective Flame Retardant for Epoxy Resin. Chin. J. Polym. Sci..

[14] Lee S., Morgan A.B., Schiraldi D.A., Maia J. (2019). Improving the flame retardancy of polypropylene foam with piperazine pyrophosphate via multilayering coextrusion of film/foam composites. J. Appl. Polym. Sci..

[15] Chen T., Xiao X., Wang J., Guo N. (2019). Fire, thermal and mechanical properties of TPE composites with systems containing piperazine pyrophosphate (PAPP), melamine phosphate (MPP) and titanium dioxide (TiO_2_). Plast. Rubber Compos..

[16] Tang W., Cao Y., Qian L., Chen Y., Qiu Y., Xu B., Xin F. (2019). Synergistic Charring Flame-Retardant Behavior of Polyimide and Melamine Polyphosphate in Glass Fiber-Reinforced Polyamide 66. Polymers.

[17] Liang C., Du Y., Wang Y., Ma A., Huang S., Ma Z. (2021). Intumescent fire-retardant coatings for ancient wooden architectures with ideal electromagnetic interference shielding. Adv. Compos. Hybrid Mater..

[18] Dong Y., Wang G., Yang J. (2013). Influences of silicone emulsion on fire protection of waterborne intumescent fire-resistive coating. J. Coat. Technol. Res..

[19] Mariappan T., Agarwal A., Ray S. (2017). Influence of titanium dioxide on the thermal insulation of waterborne intumescent fire protective paints to structural steel. Prog. Org. Coat..

[20] Yew M.C., Ramli Sulong N.H., Yew M.K. (2015). Influences of flame-retardant fillers on fire protection and mechanical properties of intumescent coatings. Prog. Org. Coat..

[21] Mustapa S., Sulong N.R. (2017). Performance of Palm Oil Clinker as a Bio-Filler with Hybrid Fillers in Intumescent Fire Protective Coatings for Steel. Sains Malays..

[22] Nasir K.M., Sulong N.R., Johan M.R., Afifi A.M. (2018). An investigation into waterborne intumescent coating with different fillers for steel application. Pigment Resin Technol..

[23] Beh J.H., Yew M.C., Saw L.H. (2020). Fire Resistance and Mechanical Properties of Intumescent Coating Using Novel BioAsh for Steel. Coatings.

[24] Wang C., Huo S., Liu S., Hu Q., Zhang Q., Liu Z. (2020). Recycle of magnesium alloy scrap for improving fire resistance, thermal stability, and water tolerance of intumescent fire-retardant coatings. J. Coat. Technol. Res..

[25] Yew M.C., Ramli Sulong N.H., Yew M.K. (2014). Investigation on solvent-borne intumescent flame-retardant coatings for steel. Mater. Res. Innov..

[26] Xie W., Chen H., He D., Zhang Y., Fu L., Ouyang J., Yang H. (2019). An emerging mineral-based composite flame retardant coating: Preparation and enhanced fireproof performance. Surf. Coat. Technol..

[27] Ullah S., Ahmad F., Shariff A.M., Bustam M. (2014). Synergistic effects of kaolin clay on intumescent fire retardant coating composition for fire protection of structural steel substrate. Polym. Degrad. Stab..

[28] Beheshti A., Heris S.Z. (2015). Experimental investigation and characterization of an efficient nanopowder-based flame retardant coating for atmospheric-metallic substrates. Powder Technol..

[29] Ullah S., Ahmad F., Yusoff P.S.M.M. (2012). Effect of boric acid and melamine on the intumescent fire-retardant coating composition for the fire protection of structural steel substrates. J. Appl. Polym. Sci..

[30] Zhou G., Li S., Zhang X., Liu Z., He M., Chen X., Yang W. (2022). Synthesis and properties of a fire-retardant coating based on intercalated expandable graphite-modified cellulose for steel structures. J. Build. Eng..

[31] Tang B., Feng W., Guo J., Sun J., Zhang S., Gu X., Li H., Yang W. (2019). Hydrophobic modification of pentaerythritol and its application in fire-retardant coatings for steel structures. Prog. Org. Coat..

[32] Li H., Hu Z., Zhang S., Gu X., Wang H., Jiang P., Zhao Q. (2014). Effects of titanium dioxide on the flammability and char formation of water-based coatings containing intumescent flame retardants. Prog. Org. Coat..

[33] Yew M.C., Ramli Sulong N.H., Yew M.K. (2014). Fire propagation performance of intumescent fire protective coatings using eggshells as a novel biofiller. Sci. World J..

[34] Sut A., Metzsch-Zilligen E., Großhauser M., Pfaendner R., Schartel B. (2019). Synergy between melamine cyanurate, melamine polyphosphate and aluminum diethylphosphinate in flame retarded thermoplastic polyurethane. Polym. Test..

[35] Wang G., Yang J. (2011). Influences of glass flakes on fire protection and water resistance of waterborne intumescent fire resistive coating for steel structure. Prog. Org. Coat..

[36] Hu Z., Zhong Z.Q., Gong X.D. (2020). Flame retardancy, thermal properties, and combustion behaviors of intumescent flame-retardant polypropylene containing(poly)piperazine pyrophosphate and melamine polyphosphate. Polym. Adv. Technol..

